# Do we need to improve the reporting of evidence in tendinopathy management? A critical appraisal of systematic reviews with recommendations on strength of evidence assessment

**DOI:** 10.1136/bmjsem-2020-000920

**Published:** 2021-02-23

**Authors:** Dimitris Challoumas, Neal L Millar

**Affiliations:** Institute of Infection, Immunity and Inflammation College of Medicine, Veterinary and Life Sciences, University of Glasgow, Glasgow, UK

**Keywords:** tendon, tendinopathy, evidence based review

## Abstract

**Objective:**

To critically appraise the quality of published systematic reviews (SRs) of randomised controlled trials (RCTs) in tendinopathy with regard to handling and reporting of results with special emphasis on strength of evidence assessment.

**Data sources:**

Medline from inception to June 2020.

**Study eligibility:**

All SRs of RCTs assessing the effectiveness of any intervention(s) on any location of tendinopathy.

**Data extraction and synthesis:**

Included SRs were appraised with the use of a 12-item tool devised by the authors arising from the Preferred Reporting Items in Systematic Reviews and Meta-Analyses statement and other relevant guidance. Subgroup analyses were performed based on impact factor (IF) of publishing journals and date of publication.

**Results:**

A total of 57 SRs were included published in 38 journals between 2006 and 2020. The most commonly used risk-of-bias (RoB) assessment tool and strength of evidence assessment tool were the Cochrane Collaboration RoB tool and the Cochrane Collaboration Back Review Group tool, respectively. The mean score on the appraisal tool was 46.5% (range 0%–100%). SRs published in higher IF journals (>4.7) were associated with a higher mean score than those in lower IF journals (mean difference 26.4%±8.8%, p=0.004). The mean score of the 10 most recently published SRs was similar to that of the first 10 published SRs (mean difference 8.3%±13.7%, p=0.54). Only 23 SRs (40%) used the results of their RoB assessment in data synthesis and more than half (n=30; 50%) did not assess the strength of evidence of their results. Only 12 SRs (21%) assessed their strength of evidence appropriately.

**Conclusions:**

In light of the poor presentation of evidence identified by our review, we provide recommendations to increase transparency and reproducibility in future SRs.

**Summary box**

**What is already known?**

- The increasing number of available treatment modalities for tendinopathy can be overwhelming to the treating healthcare professionals.
- Systematic reviews (SRs) of randomised controlled trials provide the highest level of evidence and guide application of research findings to clinical practice.
- There are several strength of evidence assessment tools used in tendinopathy SRs.
- The use of strength of evidence assessment results in data synthesis is associated with confusion and inconsistency.

**What are the new findings?**

- More than half of the SRs did not assess the strength of evidence in their results.
- Only 21% of SRs assessed their strength of evidence appropriately.
- Authors who publishing SRs on the management of tendinopathy should become familiar with strength of evidence assessment tools and apply them appropriately in their data synthesis and presentation.
- We provide recommendations to increase transparency and reproducibility in future SRs.

## Introduction

The ever-expanding arsenal of treatment regimes for tendinopathy can be overwhelming to the treating healthcare professional. New treatments continuously emerge and so do research studies that aim to assess their effectiveness. The most powerful tool that evidence-based medicine has to offer remains systematic reviews (SRs) of randomised controlled trials (RCTs), which constitute the highest level of evidence and are therefore often used to inform guidelines and guide clinical practice.^1^

Unlike a narrative review, which is purely a summary of a proportion of (or all) the available studies on a given topic without assessing the quality of the included evidence, an SR is expected to include all the relevant evidence which is comprehensively and thoroughly appraised and presented with qualitative or quantitative syntheses and accompanying strengths of evidence.^2^ For reliable, clinically relevant conclusions to be reached, the potential limitations of the included studies need to be considered.^3^ What determines the ‘quality’ of a study remains poorly defined but should relate to the extent to which its design, conduct, analysis and presentation are appropriate to answer its research question.^3^ Generally, study quality assessment includes a combined assessment of its internal validity (freedom from bias), external validity (generalisability/applicability) and precision (freedom from random error)^3^ (online supplemental figure 1). The Cochrane group, which produce some of the highest quality SRs, recommend emphasising the internal validity when assessing the quality of a study, reflected by the risk-of-bias assessment (RoB) tool they have produced.^3^ However, external validity and precision should not be overlooked.

10.1136/bmjsem-2020-000920.supp1 Supplementary data

Assessing the strength of evidence (also known as ‘certainty’, ‘quality’, ‘level’ or ‘grade’ of evidence) is closely interlinked with RoB assessment and is a process which is commonly overlooked by many SRs. Together with a meticulous methodology and comprehensive RoB assessment, this is what distinguishes a SR from other types of review articles. Assigning a strength of evidence to a finding is as important as the finding itself. Strength of evidence should accompany every assessed outcome measure within an SR for every assessed follow-up time period.^4 5^ A result with a low strength of evidence differs substantially from that with a high level of evidence in terms of its applicability to clinical practice; further research is likely to change the former but not the latter.^6^ Equally, the strength of evidence can be high in an outcome measure and low in another, and high for a certain follow-up but period and low for another.

An SR should be transparent and reproducible, and subjectivity should be kept to a minimum.^7^ Firm guidance on conducting SRs does not exist and several parameters are left to the judgement of the authors. In an attempt to optimise the quality of SRs, the Preferred Reporting Items in Systematic Reviews and Meta-Analyses (PRISMA) group have published evidence-based guidance, including a 27-item checklist.^8^ Similarly, the A MeaSurement Tool to Assess systematic Reviews (AMSTAR) group have published a critical appraisal tool for SRs aiming to help the reader/appraiser make conclusions about its quality.^9^ The aim of the former in particular is to help authors improve the reporting of SRs and meta-analyses of randomised trials and it is considered the gold standard for the authorship of SRs. However, summarising the strength of the presented evidence, which is mentioned in one of its items, does not receive sufficient attention in most SRs. Moreover, recent debate in the Lancet argues that the findings of SRs may be flawed as they often include poor-quality studies that should have not been published in the first place.^10^

Our aim was to critically appraise the quality of published SRs of RCTs in tendinopathy, with emphasis on handling and reporting of results and especially assessment of the strength of evidence, which is often overlooked. We provide recommendations for authors of future SRs aiming to optimise data synthesis, reporting and summarising the strength of evidence. The present review is also intended for readers of SRs, who need to critically appraise their quality before reaching conclusions about their findings, and finally it can be used by reviewers and editors of journals when assessing submitted SRs for publication.

## Methods

### Eligibility

Eligible articles assessed the effectiveness of any intervention(s) on tendinopathy (any type), identified themselves as an SR and/or meta-analysis and only included RCTs. Reviews including a mixture of randomised studies and other types of studies (non-randomised clinical trials, cohort studies, case series, etc) and those assessing the effectiveness of interventions on tendinopathy and other conditions were excluded. Only articles published in English were included in patients over 16 years of age. No criteria were used regarding the following parameters: publication date, journal type, type of tendinopathy and intervention, outcome measures and length of follow-up.

### Search strategy: screening

**Figure 1:**
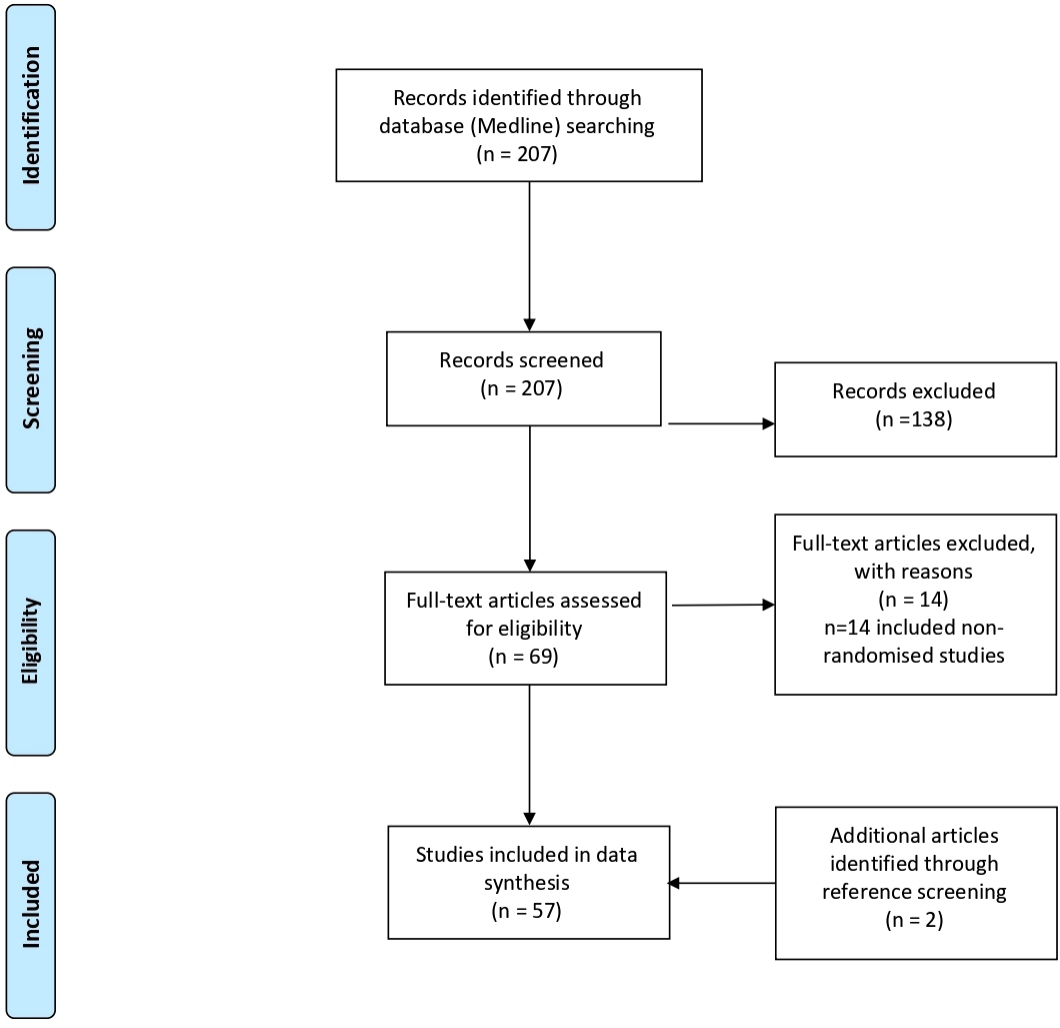
PRISMA flow diagram of included studies. PRISMA, Preferred Reporting Items in Systematic Reviews and Meta-Analyses.

A literature search was conducted by the first author via Medline in June 2020 with the following Boolean operators in ‘All Fields’: “((systematic review) OR (meta-analysis) AND (tendin*) AND (randomi*)). For all eligible articles, the reference lists and PubMed’s ‘similar articles’ list were screened to identify potentially eligible articles that may have been missed at the initial search. Figure 1 (PRISMA flow chart) illustrates the article screening process.

The initial search returned a total of 207 articles. After exclusion of non-eligible articles according to our predefined criteria and inclusion of articles identified from reference screening, 57 reviews were included in our appraisal.

### Data extraction: handling

The included articles were read by the first author and their key characteristics were tabulated in Microsoft Word. Each article was then reread and appraised with regard to the handling and reporting of results based on our prespecified appraisal tool.

### Appraisal tool: rationale and explanation of items

We developed a 12-item appraisal checklist based on guidance from the PRISMA checklist predominantly, as well as other key documents, namely the Grading of Recommendations Assessment, Development and Evaluation (GRADE) tool, the AMSTAR checklist and the Cochrane Collaboration Back Review Group.^4 5 8 9^ We highlight that our tool was created merely for the purposes of the present review, it does not intend to appraise SRs as a whole and it cannot replace other tools such as the PRISMA checklist and the AMSTAR tool; it can, however, be used in conjunction with them. It intends to assess data synthesis and presentation without considering other important methodological aspects (eg, search strategy, screening, inclusion criteria). Each item receives either a ‘0’, ‘1’ or ‘2’ except for four items (2, 4, 11, 12) which only receive ‘0’ or ‘1’. The maximum score is 20 for SRs that include both qualitative and quantitative analyses and 18 for those including one or the other. The tool is intended to be used as a checklist rather than a scoring system, however, scoring was used in the present review for the purposes of subgroup comparisons. The 12 items are divided into the following three categories and a description of how the tool was used is provided below:

#### ROB assessment

Item 1: The RoB assessment of each included RCTs, with or without an ‘overall risk’ for each study, needs to be used somehow for summarising the strength of evidence, which is its primary purpose, and this needs to be explained in the methods. This can be through subgroup analyses, for example, only synthesising results of studies with low overall RoB (which is indirectly associated with strength of evidence assessment).

Item 2; RoB should be assessed for each outcome measure separately to determine the strength of evidence of the results for that specific outcome measure. Assessing RoB on an outcome measure level rather than a study level becomes particularly important when the study includes patient-reported measures and measures assessed directly by another assessor. With the former, if the participants are not blinded then automatically the assessment of outcome measures cannot be blinded either as the assessor is the participant his/herself. For non-patient-reported measures, regardless of blinding of participants, the blinding of assessment depends solely on whether the assessor (researcher) is blinded or not. If RoB is assessed on a study and not outcome level this should be stated in the methods with a justification (ie, all outcome measures were patient reported or participants of all studies blinded, etc). For SRs including one outcome measure only, this item was not scored.

#### Pooling of results

Item 3: The principal summary measures for each included outcome measure need to be stated in the methods. For quantitative analyses, where outcome measures are continuous, a justification needs to be provided for the use of (raw) mean differences (MD) and not standardised MD (SMD) and vice versa (ie, identical or different outcome measure tools used across studies, respectively). Similarly, for dichotomous outcomes, the authors should state in the methods whether OR or relative risks (RR) were used.

Item 4: An SR without a meta-analysis should ideally have some quantitative summary measures (ie, MD, SMD, OR or RR with accompanying CI) to demonstrate the treatment effect of the assessed intervention over the comparator. Where results are pooled only based on direction of effect (ie, increased, decreased or unchanged), this needs to be stated in the methods with a justification. If pooling is not possible and results are only described narratively, the reason should be stated (usually substantial clinical heterogeneity); in such cases, we question whether the article should be identified as an SR.

Item 5: When meta-analyses are performed, the model used needs to be stated (ie, fixed effects or random effects) with a justification and the statistical heterogeneity assessment (usually χ^2^ and/or I^2^ test) and how it was used in data syntheses. Sensitivity and subgroup analyses should also be pre-defined in the methods.

Items 6 and 7: Results should only be pooled for similar follow-up time periods which should be prespecified in the methods. Traditionally these are short term, mid term and long term. The range of these should be defined by the authors in the methods based on the population, outcome measures and interventions. Equally, assessed outcome measures should be predefined (usually as part of PICOS).

Item 8: A statement needs to be included in the section explaining how the authors dealt with missing data, which are usually methodological details of the included studies needed for the RoB assessment and descriptive statistics for quantitative analyses. For both, ideally attempts should be made to contact the authors of the RCTs for retrieval of the missing data and this should be stated. If significant statistical data of included studies cannot be retrieved (ie, sample sizes and means) these studies should be excluded from quantitative analyses; if variability statistics (SD) are missing, the authors have the option to impute these using data from other RCTs and the imputation method used should be described.

#### Strength of evidence assessment

Items 9–12: Assigning a strength of evidence to a result is an essential part of an SR and it should be determined separately for each predefined outcome measure at each predefined follow-up time period. By and large this is determined based on the overall quality of the included studies and the extent of consistency of their findings with considerations of other limitations across studies. Regardless of the tool used, which should be included in the methods with a brief description, the authors should clearly describe how data were synthesised with regard to assessing the strength of evidence, stating how RoB assessment was used and what other paramaters/limitations were considered. Excluding the GRADE tool, which has clear instructions on how specific intrastudy (RoB) and interstudy (inconsistency, imprecision, indirectness, publication bias) limitations should be handled, the authors should clarify: (1) whether study quality was determined based on RoB only or whether other parameters were taken into account (eg, precision, external validity) with a justification; (2) how consistency was defined (ie, based on direction of effect, overlap of CIs, etc). When there is some clinical heterogeneity across studies but not sufficient to preclude pooling of results, this should be accounted for and used as part of the strength of evidence assessment. More specifically, authors should include a statement to show that they have considered/assessed for potential differences in included populations, interventions and outcome measures used across studies and how they think this might affect the strength of the evidence of their results. Finally, a statement in the methods should be included to acknowledge the risk of publication bias and whether it was assessed formally. A funnel plot should be constructed and inspected/accompanied with the appropriate statistical tests (ie, Egger’s test) where the results of a considerable number of studies (usually 10 or more) were pooled. For SRs including one outcome measure only, item 11 was not scored.

## Statistical analysis: subgroup analysis

A mean score was calculated for all appraised SRs. Subgroup analyses included comparisons of mean scores of reviews published in a higher versus a lower impact factor (IF) journal (mean IF of included journals used as cut-off). The latest journal IFs were obtained from the journal’s official website. For journals without an IF, a score of 0 was given. Finally, the mean score of the first 10 published SRs was compared with that of the last 10 published. All comparisons were performed with two-tailed independent samples t-tests in Graphpad Prism V.8. Significance levels were set at p<0.05.

## Results

A total of 57 articles published in 38 journals were included in our review.^11–67^ Publication date ranged from 2006 to 2020, journal IFs ranged from 0 to 59.102 (mean 4.7, median 2.55). The journal with the most published SRs of RCTs on tendinopathy interventions was the *British Journal of Sports Medicine* (n=6) and the most commonly investigated tendinopathy in isolation was shoulder (calcific and non-calcific; n=24), followed by lateral elbow (n=10) and patellar (n=8). A total of 10 reviews assessed tendinopathy as a generic condition and assessed the same intervention(s) on more than one location of tendinopathy and two reviews included RCTs of ‘lower limb tendinopathies’. The most frequently assessed intervention in isolation was extracorporal shock-wave therapy (n=10), followed by platelet-rich plasma (n=7), however, several SRs included more than one ‘related’ intervention (ie, ‘non-surgical therapies’, ‘conservative treatments’, ‘injection therapies’, etc).

Online supplemental table 1 provides an overview of the included SRs with relevant characteristics and a summary of their key findings.^11–67^ Table 1 shows our appraisal tool and the results of the appraisal for each SR with overall scores both for each SR and for each item (explanation of the items in table caption).^11–67^

10.1136/bmjsem-2020-000920.supp2 Supplementary data

**Table 1:** Appraisal results of each study using our tool with total scores and percentages both for each study and for each tool item

Study	Risk of bias (RoB) assessment		Pooling of results						Strength of evidence assessment				Total score	%
Item	1 (0–2)	2 (0–1)	3 (0–2)	4 (0–1)	5 (0–2)	6 (0–2)	7 (0–2)	8 (0–2)	9 (0–2)	10 (0–2)	11 (0–1)	12 (0–1)		
Arirachakaran *et al* 11	0	0	2	1	–	2	0	0	0	2	0	0	7/18	39
Arirachakaran *et al* 12	0	0	2	1	0	2	0	0	0	2	0	0	7/20	35
Balasubramaniam 13	2	0	2	1	2	–	0	2	2	0	0	0	11/18	61
Bannuru *et al* 14	2	0	2	1	2	–	0	0	0	0	0	0	7/18	39
Bjordal *et al* 15	2	0	2	1	–	2	0	0	0	2	0	0	9/18	50
Boudreault *et al* 16	0	0	2	0	–	2	0	0	1	2	0	0	7/18	39
Buchbinder *et al* 17	2	0	2	1	2	2	2	2	2	0	1	1	17/20	85
Catapano *et al* 18	0	0	2	0	0	–	0	0	0	0	0	0	2/18	11
Challoumas *et al* 19	2	0	0	1	2	–	2	0	2	0	1	1	11/18	61
Challoumas *et al* 20	2	0	0	1	2	–	2	0	2	0	1	1	7/11	61
Chen *et al* 21	0	0	2	1	–	2	0	2	0	2	0	0	9/18	50
Coombes *et al* 22	2	0	2	1	2	2	2	2	2	2	0	1	17/20	85
Dan *et al* 23	2	1	2	1	2	2	2	2	2	2	1	1	20/20	100
de Vos *et al* 24	2	0	0	1	1	–	0	0	2	0	0	0	6/18	33
Desjardins-Charbonneau *et al* 25	0	0	2	1	0	2	0	2	0	2	0	0	9/20	45
Desjardins-Charbonneau *et al* 26	0	0	2	0	0	2	0	2	1	2	0	0	9/20	45
Desmeules *et al* 27	0	0	0	1	0	–	0	0	1	0	0	0	2/18	11
Desmeules *et al* 28	0	0	0	0	0	–	0	0	0	0	0	0	0/18	0
Desmeules *et al* 29	0	0	2	0	0	2	0	0	1	0	0	0	5/20	25
Dong *et al* 30	2	–	2	1	–	2	1	0	0	0	–	0	8/16	50
Dupley and Charalambous 31	0	–	2	1	–	2	1	0	0	0	–	0	6/16	38
Fitzpatrick *et al* 32	1	–	2	1	–	2	2	2	1	0	–	0	11/16	69
Haslerud *et al* 33	2	0	2	1	–	2	0	2	1	0	0	0	10/18	56
Heales *et al* 34	2	0	2	1	2	–	0	2	2	0	1	1	13/18	72
Huisstede *et al* 35	2	0	0	1	0	–	2	0	2	0	0	0	7/18	39
Ioppolo *et al* 36	0	0	0	1	0	0	0	0	0	0	0	0	1/20	5
Krey *et al* 37	0	0	0	0	0	–	0	0	0	0	0	0	0/18	0
Lafrance *et al* 38	0	0	2	1	0	–	1	2	1	0	0	0	9/18	50
Larsson *et al* 39	1	0	0	0	0	–	0	0	2	0	0	0	3/18	17
Lee *et al* 40	1	0	0	0	0	–	2	0	2	0	0	1	6/18	33
Li *et al* 77	0	0	2	0	–	2	1	2	0	0	0	0	7/18	39
Liao *et al* 41	0	0	2	1	–	2	2	0	0	2	0	0	9/18	50
Lin *et al* 42	2	0	2	1	–	2	2	2	0	2	0	0	13/18	72
Lin *et al* 43	2	1	2	1	–	2	2	2	0	2	0	0	14/18	78
Lin *et al* 44	1	0	2	1	–	2	2	0	0	2	0	0	10/18	56
Littlewood *et al*^45^	2	0	0	1	0	–	1	0	2	0	0	0	6/18	33
Louwerens *et al* 46	1	0	2	1	0	2	1	2	2	0	1	1	14/20	70
Maffulli *et al* 47	0	0	0	0	0	–	0	0	0	0	0	0	0/18	0
Magnussen *et al* 48	0	0	0	1	0	–	0	0	0	0	0	0	1/18	6
Martimbianco *et al* 49	2	0	2	1	–	2	1	2	2	2	1	1	16/18	89
Mendonça *et al* 50	2	0	2	1	–	2	2	0	2	2	1	1	15/18	83
Miller *et al* 51	0	0	2	1	–	2	2	0	1	2	0	0	10/18	56
Mohamadi *et al* 52	2	–	2	1	2	2	2	2	0	2	–	0	15/18	83
Murphy *et al* 53	2	–	2	1	–	2	0	2	2	2	–	1	14/16	88
Nogueira and Moura 54	0	0	0	0	0	–	0	0	0	0	0	0	0/18	0
Ortega-Castillo and Medina-Porqueres 55	2	0	2	1	2	–	0	0	2	0	0	0	9/18	50
Sussmilch-Leitch *et al* 56	2	0	2	1	2	2	1	2	0	0	0	0	13/20	65
Tsikopoulos *et al* 57	0	0	2	1	2	–	2	2	0	2	0	0	11/18	61
Toliopoulos *et al* 58	0	0	0	0	0	–	0	0	1	0	0	0	1/18	6
Vander Doelen and Jelley 59	0	0	0	1	0	–	0	0	0	0	0	0	1/18	6
Verstraelen *et al* 60	0	0	2	1	2	2	2	2	0	2	0	0	13/20	65
Wasielewski and Kotsko 61	0	0	2	1	2	–	0	0	0	0	0	0	5/18	28
Woodley *et al* 62	2	0	2	1	2	2	1	2	2	0	0	1	15/20	75
Wu *et al* 63	0	0	2	1	–	2	2	2	0	2	0	0	11/18	61
Xiong *et al* 64	2	0	2	1	–	2	0	0	0	0	0	0	7/18	39
Yan *et al* 65	0	0	2	1	–	1	1	0	0	0	0	0	5/18	28
Zhang *et al* 66	0	0	2	1	2	2	0	2	0	2	0	0	11/20	55
Overall mean score	–	–	–	–	–	–	–	–	–	–	–	–	–	46.5
Overall Total for item	53/114	2/52	82/114	45/57	33/74	65/68	46/114	48/114	45/114	44/114	8/52	12/57		
%	47%	4%	72%	79%	45%	96%	40%	42%	39%	39%	15%	21%		

Item 1—was RoB assessment used in data synthesis and was it prespecified/explained in methods? (‘0’ for neither, ‘2’ for both, ‘1’ if used but not prespecified)—(PRISMA items 12, 19). Item 2—was RoB assessment performed on an outcome measure level? If not, do the authors state why in methods? (‘1’ if either fulfilled, ‘0’ if neither)—(PRISMA items 12, 19). Item 3—are principally summary measures predefined in methods and used in results? (‘0’ for neither, ‘2’ for both, ‘1’ if used but not prespecified)—(PRISMA item 13). Item 4—was the outcome measures of the SR predefined in methods? (‘0’ for no, ‘1’ for yes)—(PRISMA item 11). Item 5—is qualitative synthesis appropriate and was it prespecified/explained in methods? (‘0’ for neither or if only narrative description without pooling of results, ‘2 for both’, ‘1’ if appropriate but not prespecified or if prespecified but not done)—(PRISMA items 13, 14). Item 6—is quantitative synthesis appropriate and was it prespecified/explained in methods? (‘0’ for neither, ‘2’ for both, ‘1’ if appropriate but not prespecified)—(PRISMA items 20, 21). Item 7—was follow-up time points for data analyses predefined and used? (‘0’ for neither, ‘2’ for both, ‘1’ if used but not prespecified)—(Cochrane Back Review Group). Item 8—was missing data dealt with appropriately and was this prespecified in methods? (‘0’ for neither, ‘2’ for both, ‘1’ if appropriate but not prespecified)—(Cochrane Back Review Group). Item 9—was assessment of the strength of the evidence used and was it prespecified/explained in methods? (‘0’ for neither, ‘2’ for both, ‘1’ if used but not prespecified)—(PRISMA item 24). Item 10—was the risk of publication bias acknowledged in methods and was it assessed where appropriate (‘0’ for neither, ‘2’ for both, ‘1’ if done but not prespecified)—(PRISMA items 15, 25). Item 11—was the strength of the evidence assessed on an outcome measure level? (‘1’ for yes, ‘0’ for no or if assessment of strength of evidence not performed)—(GRADE). Item 12—was the strength of the evidence assessed appropriately according to the prespecified tool? (‘1’ for yes, ‘0’ for no or if not performed/prespecified) (Strength of evidence assessment tools used).

GRADE, Grading of Recommendations Assessment, Development and Evaluation; PRISMA, Preferred Reporting Items in Systematic Reviews and Meta-Analyses; SR, systematic review.

The most commonly used RoB assessment tools in the included SRs were the Cochrane Collaboration tool^3 67^ (n=30) and the PEDro scale^68 69^ (n=14). Of the 52 SRs that conducted an RoB assessment, only 23 (44%) explained how that would be used in data synthesis and 27 (52% reviews did not use their RoB assessment results in data synthesis at all. Where assessment of strength of evidence was predefined and applied, the most frequently used tools were the Cochrane Back Review Group tool^4^ (n=10) and the GRADE tool^5^ (n=6). Other tools for assessing strength of evidence used included the National Health and Medical Research Council (NHMRC) tool^70^ and that devised by the Cochrane musculoskeletal group.^71^ More than half of the included reviews (n=30; 53%) did not use or even mention quality/strength/level of evidence in their manuscript, while another nine included an arbitrary statement of a level of evidence in their discussion or conclusion without explaining the process of determining this anywhere in the manuscript. Of the remaining 18 reviews which described the use of a tool to assess the strength of evidence in their methods, only 12 of them used it appropriately according to the criteria/instructions of the tool (and to a lesser extend the judgement of the authors of the present review).

The items of our tool with the lowest overall score were item 2 (2/52; 4%) and item 11 (8/52; 15%), which both relate to whether assessments (RoB and strength of evidence) were conducted on an outcome measure and not study level. Those with the highest were item 6 (65/68; 95%) and item 4 (45/57; 79%).

**Figure 2:**
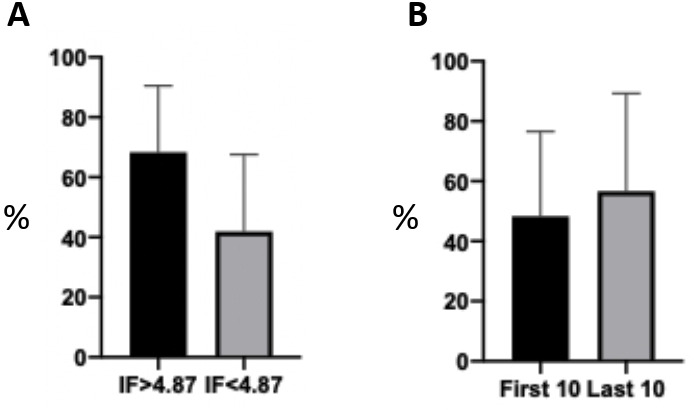
Subgroup analyses comparing the mean scores of reviews published in a higher versus a lower impact factor (IF) journals. Those published in higher if journals (>4.7; n=10) had a significantly higher mean score than those (n=47) in lower if journals (68.3% vs 41.8%, difference 26.5%±8.8%, p=0.004) (A). The first 10 published SRs (2006–2012) had a slightly lower mean score (48.3%) than the 10 most recently published ones (2019–2020; 56.6%) with a statistically insignificant difference (8.3%±13.7%, p=0.54; B). SRs, systematic reviews.

The mean score of the included SRs on our appraisal tool was 46.5% (range 0%–100%). Subgroup analyses showed that those published in higher IF journals (>4.7; n=10) had a significantly higher mean score than those (n=47) in lower IF journals (68.3% vs 41.8%, difference 26.5%±8.8%, p=0.004) (figure 2A). The first 10 published SRs (2006–2012) had a slightly lower mean score (48.3%) than the 10 most recently published ones (2019–2020; 56.6%) with a statistically insignificant difference (8.3%±13.7%, p=0.54; figure 2B).

## Discussion

Having critically analysed several published SRs of RCTs in the management of tendinopathy, we found that the results of more than half of the included SRs were presented without an accompanying strength of evidence assessment, which is as important as the results themselves for guiding clinical practice. Additionally, a substantial proportion of results from the included reviews were presented with levels of evidence assigned to them arbitrarily without described and reproducible methods. Our findings imply that the majority of SR authors may not be familiar with conducting a strength of evidence assessment and its practical implementation. The other interesting finding was that half of the reviews that performed an RoB assessment did not make any use of it; we assume this is because they either do not understand the purpose of RoB assessment and they only conducted an RoB assessment knowing that it is a prerequisite for an SR, or they left the interpretation of the link between their RoB assessment and the undetermined strength of evidence open to the reader.

As an example of the disparity in assessing the strength of evidence, a recent SR of SRs on the clinical management of tendinopathy summarised all the available evidence concluding that eccentric exercise with or without other treatments is the best available intervention.^72^ Unlike our appraisal which yielded poor overall results, their assessment of quality of the included SRs was overall high. This is predominantly because the appraisal tool they used (AMSTAR) does not take into account the importance of determining the strength of evidence for the findings. Additionally, having used very similar criteria to our review, performing searches in many more databases, and even having included some SRs with a mixture of randomised and non-randomised studies, they only included 25 SRs as opposed to 57 included in ours.

The widely used PRISMA checklist is regarded as the gold standard for the conduct and reporting of SRs.^8^ Its 27 items cover all sections of an SR, including the title (1 item), abstract (1 item), introduction (2 items), methods (12 items), results (7 items), discussion (three items) and funding (1 item). The purpose of the checklist, as its name suggests, is to increase transparency in the reporting of SR and meta-analyses, however, the conduct of some of its items remains very subjective. This is especially true for the assessment of strength of evidence, which, according to the checklist, should be performed for each outcome measure in the discussion section, however, further guidance/instructions are not provided.^8^ We argue that details of the process of strength of evidence assessment should be included in the methods section of an SR to increase transparency and reproducibility.

### Recommendations

In the following paragraphs, we provide recommendations for what we believe are the most subjective and poorly understood aspects of an SR, aiming to increase transparency, reproducibility and hence the quality of the SR and the confidence in its findings. According to the National Institute for Health Research, authors should only identify their article as an SR ‘when the account of the search, appraisal and synthesis methods would, in theory, permit the replication of the review by others’.^7^ All recommendations arise from key documents guiding the conduct of SRs: the PRISMA checklist, the Cochrane handbook, the GRADE handbook and the Cochrane Collaboration Back Review Group guidance.^4 8 67 73^

#### RoB assessment

We highlight once again that an RoB assessment is the most important part of but not exactly synonymous to study quality assessment. Where the strength of evidence assessment tool used relies on study quality (eg, Cochrane Back Review Group tool), the authors should clarify if they only use RoB assessment as an extension of study quality. Finally, RoB assessment should be performed on an outcome measure level, not a study level, especially when the study includes both patient-reported outcomes and outcomes directly measured by study faculty.

We recommend the use of the revised RoB assessment tool recently published by the Cochrane Collaboration (RoB 2) as it is more standardised and reproducible with clear instructions on how it should be used and less subjectivity.^74^ Most importantly, the creators include instructions for determination of overall RoB for each study which will substantially increase its correct implementation in data syntheses in SRs.^74^ Alternatively, the RCT methodology checklist published by the Scottish Intercollegiate Guideline Network^75^ could also be used, especially where the Cochrane Collaboration Back Review Group tool is used for strength of evidence assessment (see below). Importantly, the checklist finishes with an ‘overall assessment of the study’, which is the equivalent of RoB 2’s ‘overall RoB’ and can be used directly for strength of evidence assessment.

#### Pooling of results

All SRs should include pooling of results to make conclusions about an overall treatment effect where clinical heterogeneity allows.^8^

In the absence of significant clinical heterogeneity, results should be pooled quantitatively where there are two or more studies assessing the same interventions, ideally with pairwise meta-analyses that improve precision.^8 67^ In the context of tendinopathy interventions, unless the settings, participants and interventions are almost identical (clinical homogeneity), we suggest the use of a random-effects and not a fixed-effects model for meta-analyses.^67^ Alternatively, if the authors of the review believe that the included studies are largely homogenous clinically, the choice of the model should be made on the basis of statistical heterogeneity, which needs to be quantified with a statistical test (eg, fixed-effects for I^2^ <50%, random-effects for I^2^ >50%).^76^ Forest plots should be presented for each comparison with their accompanying MD/SMD (continuous outcomes) or OR/RR (dichotomous outcomes) and 95% CI, p value and heterogeneity test (eg, I^2^ statistic).^8 67^ In the presence of significant statistical heterogeneity (eg, >50%), attempts should be made through sensitivity analyses for a single study that may be responsible for the high heterogeneity to be identified and removed from the comparison before the analysis is rerun.^67 76^ No more than one study should be removed. Where the I^2^ test is substantial (>75%) despite sensitivity analyses, the meta-analysis should be abandoned.^76^

For qualitative reviews, where numerical values of treatment effects cannot be obtained and pooled quantitatively, pooling should be performed based on direction of effect for studies assessing the same interventions, even in the presence of some clinical heterogeneity, which should be recognised and accounted for in the strength of evidence assessment.^4^ The statistical significance test result reported by the authors of the RCT for intergroup differences should be accounted for when pooling results (ie, a statistically insignificant difference between the two groups will count as ‘no difference’). A table with pooled results and accompanying strengths of evidence should be used to make the pooling clearer to the reader (eg, table 1).

Data should only be combined for similar, predefined follow-up time periods, that is, short term, mid term and long-term.^4^ These periods should ideally be subdivided especially when their range is large and when there are sufficient data for pooling. For instance, for a predefined ‘short-term follow-up’ at 0–12 weeks, this can be subdivided into early short term (eg, 2–6 weeks) and late short term (7–12 weeks).

#### Strength of evidence assessment

The two tools most frequently used in our included SRs (Cochrane Back Review Group and GRADE) differ substantially and we advocate the preferential use of one over the other in specific situations.^4 73^ Unlike the Cochrane Back Review Group tool, the intention of which is to allocate a ‘level’ (strength) of evidence to data pooled qualitatively, the GRADE tool is very comprehensive, it considers limitations across studies, not only within studies and is very transparent.^4 73^ The main difference of the two tools lies within the fact that the Cochrane Back Review Group tool relies on quality assessment of each study; the GRADE tool advocates assessing the quality of the body of the pooled evidence, not individual studies. The former is primarily based on direction of effect (ie, qualitative synthesis of results) and the latter on quantitative analyses.

Considering the nature and the intention of these two tools, we recommend the use of the GRADE tool for all SRs where comparisons of interventions include any type of quantitative analyses (with or without a meta-analysis) and the Cochrane Back Review Group where only direction of effect is used for pooling of results (ie, qualitative analysis only). However, when the Cochrane Back Review Group tool is used, in addition to RoB assessment which should be used to determine the level of evidence for each outcome measure, we recommend using imprecision, indirectness of evidence and publication bias (where appropriate) to downgrade the level of evidence in a similar fashion to the GRADE tool. Besides, van Tulder *et al* state in their original publication that in addition to methodological quality of the original studies, participants, interventions and outcomes should also be taken into account when levels of evidence are attributed to qualitative analyses.^4^ Table 2 and table 3 can be used to increase transparency of the methods used for assessing the strength of evidence. Alternatively, the ‘summary of findings’ table suggested by GRADE can be used, separately for each outcome measure and follow-up time period with explanations for each downgrading of the evidence.^73^

**Table 2:** Suggested pooling of results for qualitative analyses (example)

Comparison	Follow-up	Study	Outcome measure 1	Outcome measure 2	Outcome measure 3
Intervention 1 versus intervention 2	Short/mid/long term	Author (year)	↓	↓	↓
		Author (year)	↓	↔	↓
		Author (year)	↓	↑	–
Overall intervention 1 versus intervention 2 (evidence level)			↓ (moderate)	- (conflicting)	↓ (limited)

**Table 3:** Suggested description of strength of evidence assessment

Comparison	Outcome measure	Follow-up	Overall RoB	Inconsistency	Imprecision	Indirectness	Publication bias	Strength of evidence
Intervention_1_ vs Intervention_2_	Outcome measure 1	Short term	High/low/unclear	High/low risk	High/low risk	High/low risk	High/low risk	High/moderate/low (very low, limited, conflicting)
		Mid term	High/low/unclear	High/low risk	High/low risk	High/low risk	High/low risk	High/moderate/low (very low, limited, conflicting)
		Long term	High/low/unclear	High/low risk	High/low risk	High/low risk	High/low risk	High/moderate/low (very low, limited, conflicting)
	Outcome measure 2	Short term	High/low/unclear	High/low risk	High/low risk	High/low risk	High/low risk	High/moderate/low (very low, limited, conflicting)
		Mid term	High/low/unclear	High/low risk	High/low risk	High/low risk	High/low risk	High/moderate/low (very low, limited, conflicting)
		Long term	High/low/unclear	High/low risk	High/low risk	High/low risk	High/low risk	High/moderate/low (very low, limited, conflicting)
	Outcome measure 3	Short term	High/low/unclear	High/low risk	High/low risk	High/low risk	High/low risk	High/moderate/low (very low, limited, conflicting)
		Mid term	High/low/unclear	High/low risk	High/low risk	High/low risk	High/low risk	High/moderate/low (very low, limited, conflicting)
		Long term	High/low/unclear	High/low risk	High/low risk	High/low risk	High/low risk	High/moderate/low (very low, limited, conflicting)

RoB, risk of bias.

Neither the GRADE tool nor the Cochrane Collaboration Back Review Group tool come without disadvantages.^4 5^ Despite GRADE’s comprehensiveness, which includes a detailed handbook providing explanation and instructions of all the steps, its conduct requires decisions that are based on the assessor’s judgement, for example, determining whether the overall RoB for an outcome measure is high or low when pooling studies with overall high and low RoB and deciding whether the magnitude of inconsistency and indirectness is significant enough to justify downgrading the evidence.^73^ Nevertheless, we believe that it is the most thorough and transparent tool for strength of evidence assessment and it should be used preferentially over other tools where possible. Similarly, the Cochrane Collaboration Back Review Group tool requires subjective decisions for the determination of the strength of evidence for a result, primarily due to its lack of definition for its two main criteria; (1) consistency of results and (2) study quality. Additionally, unlike GRADE, it does not consider ‘between-study’ limitations, such as imprecision of results and indirectness of evidence.

An SR is no small undertaking and should not make for an easy publication. Future authors who only intend to summarise the available evidence without a comprehensive quality assessment and strength of evidence assessment should strongly consider identifying their reviews as narrative, not systematic. Admittedly, strict word counts and other restrictions from journals undoubtedly have a negative influence on the quality of the published SRs; we, therefore, invite the editors of publishing journals to either loosen restrictions for submitted articles or discuss word counts and accompanying material (tables/figures) with authors on a case-by-case basis. Publication of narrative reviews should also become an option in all publishing journals.

## Conclusions

In the present review, we have demonstrated that the majority of SRs of RCTs in tendinopathy do not assess the strength of evidence of their results and this can substantially influence their application to clinical practice. In the future, authors implicated in producing and publishing SRs on the management of tendinopathy should become familiar with strength of evidence assessment tools and apply them appropriately in their data synthesis and presentation. Finally, relevant guidance documents should emphasise the importance of strength of evidence assessment and provide more detailed instructions for its conduct to increase consistency and transparency.

10.1136/bmjsem-2020-000920.supp3 Supplementary data
