# Supplementary material for: Do we need to improve the reporting of evidence in tendinopathy management? A critical appraisal of systematic reviews with recommendations on strength of evidence assessment
Source: BMJ Open Sport Exerc Med. 2021 Feb 23;7(1):e000920. doi: 10.1136/bmjsem-2020-000920 (PMC7907875; doi:10.1136/bmjsem-2020-000920)
Supplement: Supplementary data [file bmjsem-2020-000920supp001.pdf]

Supplementary Figure 1: Useful definitions

Study quality: the extent to which its design, conduct, analysis and presentation were appropriate to answer its research question. Consists of internal validity, external validity and precision

Quality/strength of evidence: one's confidence in the estimates of effect. This is usually "high", "moderate", "low", "very low", "limited" or "conflicting" depending on the grading tool used

Internal validity of a study: freedom from methodological bias (extent to which confounding factors were minimised); i.e. did the study use a sound methodology?

External validity of a study: the extent to which the results of the study are applicable and generalisable to the target population in clinical practice. This depends on the population, interventions and outcome measures used in studies and how they relate to those in clinical practice.

Precision: freedom from random error, i.e. the likelihood of the results of the studies to represent the true effect of the tested intervention. Generally, the smaller the population and the fewer the events in a study, the greater the imprecision (reflected by a wider confidence interval). Similarly, in the context of a systematic review/meta-analysis, the wider the confidence interval of the estimate of the treatment effect the higher the imprecision of the pooled evidence.

Directness of evidence: evidence arising from direct comparisons of interventions, delivered to the populations in which we are interested, and measures the outcomes important to patients. The four sources of indirectness are: differences in populations, differences in interventions, differences in outcome measures (surrogate outcomes) and indirect comparisons. Indirectness of evidence is synonymous to clinical

heterogeneity when it is assessed on an “across studies” (“inter-study”) level and external validity when on a “within studies” (“intra-study”) level.

Consistency of results: the extent to which all included studies have the same underlying effect. In the context of a systematic review/meta-analysis, inconsistency of results across studies is synonymous to statistical heterogeneity, which is usually quantified with the I^2 statistic. The I^2 statistic quantifies the proportion of the variation in point estimates due to among-study differences.
