# Supplementary material for: Do we need to improve the reporting of evidence in tendinopathy management? A critical appraisal of systematic reviews with recommendations on strength of evidence assessment
Source: BMJ Open Sport Exerc Med. 2021 Feb 23;7(1):e000920. doi: 10.1136/bmjsem-2020-000920 (PMC7907875; doi:10.1136/bmjsem-2020-000920)
Supplement: Supplementary data [file bmjsem-2020-000920supp002.pdf]

Authors	Journal	Tendinopath	Numbe	Intervention	Outcome	Risk of	Strength of	Summary of	Meta-

	(Impact Factor)	y	r of include d studies	Assessed	Measures	Bias Assessme nt Tool	Evidence Assessment Tool	Findings	analys is
**Arirachakaran et al. (2017)[11]**	J Orthop Traumatol (1.273)	Lateral Elbow	10	PRP, Autologous blood, corticosteroid injection	Pain, function, pain pressure threshold	Cochrane	None	PRP van improve pain and fewer complications. Autologous blood can improve pain, function and pain pressure thresholds but has higher complication rates.	Yes
**Arirachakaran et al. (2017)[12]**	Eur J Orthop Surg Traumatol (1.560)	Shoulder calcific	7	ESWT, US-guided lavage, corticosteroid injection and combined treatment	Pain, function	Cochrane	None	US-guided lavage is the treatment of choice	Yes
**Balasubramanian et al. (2015)[13]**	Phys Sportsmed (1.874)	All	9	PRP	Pain, disability	Furlan et al. (2009)	Cochrane Back Review Group	PRP looks promising but more research needed	No
**Bannuru et al. (2014)[14]**	Ann Intern Med	Shoulder calcific	28	ESWT	Pain, function,	Cochrane	None	High-energy ESWT is	No

	(19.315)				resolution of calcifications			effective at improving pain and function	
**Bjordal et al. (2008)**[15]	BMC Musculoskelet Disord (2.050)	Lateral Elbow	18	Laser therapy	Pain, global health, function, pain pressure threshold, sick leave	PEDro	None	Laser therapy administered with optimal doses can provide short-term pain relief and improve disability	Yes
**Boudreault et al. (2014)**[16]	J Rehabil Med (1.907)	Shoulder	12	Oral NSAIDs	None defined	Cochrane	None	Oral NSAIDs effective at reducing short-term pain but not function	Yes
**Buchbinder et al. (2006)**[17]	J Rheumatol (3.187)	Lateral Elbow	9	ESWT	Pain, function (not pre-defined)	Tool devised by authors	Cochrane Musculoskeletal Group	ESWT provides little to no benefit in pain and function	Yes
**Catapano et al. (2020)**[18]	PMR (1.902)	Shoulder	5	Dextrose Prolotherapy	None defined	Cochrane	None	Prolotherapy is potentially useful adjunct to physical therapy	No
**Challoumas et al. (2019a)**[19]	BMJ Open Sport Exerc Med (1.510)	All	12	Surgery	Pain, function, ROM, treatment	Cochrane	Cochrane Back Review Group	Surgery superior to no treatment/placebo but not	No

					success			sham surgery or physiotherapy	
**Challoumas et al. (2019b)[20]**	Br J Sports Med (11.645)	All	10	Topical GTN	Pain, tenderness, function, strength, ROM, patient satisfaction	Cochrane	Cochrane Back Review Group	Topical GTN superior to placebo in medium term	No
**Chen et al. (2019)[21]**	Arthroscopy (4.433)	Patellar	11	Non-surgical treatments	Pain, function	PEDro	None	LR-PRP is most effective non-surgical treatment	Yes
**Coombes et al. (2010)[22]**	Lancet (59.102)	All	41	Corticosteroid and other injections	Pain, function, improveme nt	PEDro	Cochrane Back Review Group	Corticosteroid injections are effective in the short-term, other injections may provide long-term benefit for lateral elbow tendinopathy	Yes
**Dan et al. (2019)[23]**	Cochrane Database Syst Rev (7.755)	Patellar	2	Surgery	Pain, function, satisfaction, QoL, withdrawals , adverse events,	Cochrane	GRADE	Inconclusive due to low quality of evidence; surgery likely no more effective than eccentric	Yes

					tendon rupture, return to sport			exercise	
**de Vos et al. (2014)[24]**	Br J Sports Med (11.645)	Lateral Elbow	6	PRP	Pain, function	PEDro	Cochrane Back Review Group	PRP not effective	No
**Desjardins-Charbonneau et al. (2015a)[25]**	Int J Sports Phys Ther (2.55)	Shoulder	10	Taping	Pain, function, ROM, strength	Cochrane	None	Inconclusive due to low quality of evidence	Yes
**Desjardins-Charbonneau et al. (2015b)[26]**	J Orthop Sports Phys Ther (3.058)	Shoulder	21	Manual therapy	Pain, function	Cochrane	None	Manual therapy may decrease pain but it is unclear if it improves function	Yes
**Desmeules et al. (2016a)[27]**	J Occup Health (1.800)	Shoulder	10	Exercise	Work-related outcomes	Cochrane	None	Exercise is effective at treating workers and promotes return to work	No
**Desmeules et al. (2016b)[28]**	Physiothera py (2.534)	Shoulder	6	TENS	Pain, ROM (not pre-defined)	Cochrane	None	Inconclusive due to low quality of evidence	No
**Desmeules et al. (2015)[29]**	Phys Ther Sport (2.000)	Shoulder	11	Therapeutic US	Pain, function	Cochrane	None	Therapeutic US administered with exercise no	Yes

								more superior than exercise alone. Compared to laser treatment it is less effective at alleviating pain	
**Dong et al. (2016)[30]**	Br J Sports Med (11.645)	Lateral Elbow	27	Injection therapies	Pain	Cochrane	None	Some injection therapies can be effective (e.g. BOTOX and PRP) but not corticosteroids. Hyaluronate and prolotherapy need more research.	Yes
**Dupley & Charalambous (2017)[31]**	Knee Surg Relat Res (No IF)	Patellar (refractory)	2	PRP	Function	None	None	PRP superior to dry needling and ESWT	Yes
**Fitzpatrick et al. (2017)[32]**	Am J Sports Med (6.093)	All	18	PRP	Pain	Cochrane	None	Good evidence to support single injection of PRP under US guidance	Yes
**Haslerud et al. (2015)[33]**	Physiother Res Int (1.33)	Shoulder	17	Laser therapy	Pain, global improveme nt, function	PEDro	None	Laser therapy can offer clinically	Yes

								relevant pain relief and improvement in symptoms alone and in combination with physiotherapy	
**Heales et al. (2020)[34]**	Musculoskelet Sci Pract (2.319)	Lateral Elbow	7	Forearm and wrist orthoses	Pain, function	Joanna Briggs Institute	GRADE	Orthoses can improve pain and pain-free grip strength but not maximal grip strength	No
**Huisstede et al. (2011)[35]**	Man Ther (2.158)	Shoulder (calcific and non-calcific)	17	ESWT	Pain, function, recivery	Furlan et al. (2009)	Cochrane Back Review Group	High-energy ESWT effective for calcific but not non-calcific tendinopathy	No
**Ioppolo et al. (2013)[36]**	Arch Phys Med Rehabil (2.697)	Shoulder calcific	6	ESWT	Clinical improvement (function), resorption of deposits	PEDro	None	ESWT effective in terms of pain, function and resorption of calcific deposits	Yes
**Krey et al. (2015)[37]**	Phys Sportsmed (1.874)	All	4	Tendon needling	None defined	None	None	Tendon needling and possible PRP improve patient-	No

								reported outcome measures	
**Lafrance et al. (2019)**[38]	BMJ Open Sport Exerc Med (1.510)	Shoulder calcific	3	US-guided lavage	Pain, function, calcification size	Cochrane	None	Ultrasound-guided lavage is more effective than shockwave therapy or a corticosteroid injection alone	No
**Larsson et al. (2012)**[39]	Knee Surg Sports Traumatol Arthrosc (3.149)	Patellar	13	All	None defined or used	Cochrane Back Review Group	Cochrane Back Review Group	Eccentric training most effective, US therapy not effective. Surgery, sclerosing injections and ESWT should be further investigated	No
**Lee et al. (2011)**[40]	J Should Elb Surg (2.865)	Shoulder calcific	9	ESWT	Pain, function	PEDro	NHMRC	Inconclusive due to low quality of evidence	No
**Li et al. (2019)**[41]	Medicine (Baltimore) (1.870)	Lateral Elbow	7	PRP, corticosteroid injection	Pain, function	Cochrane	None	Corticosteroid injection superior to PRP in short-term	Yes

								but PRP more effective in long-term	
**Liao et al. (2018)[42]**	Am J Phys Med Rehabil (1.908)	Lower limb tendinopathies	29	ESWT	Pain, function	PEDro	None	ESWT is effective for pain and function	Yes
**Lin et al. (2020)[43]**	Diagnostics (Basel) (2.489)	Shoulder	5	PRP	Pain, function	Cochrane	None	PRP may be beneficial for long-term pain	Yes
**Lin et al. (2019)[44]**	Arch Phys Med Rehabil (2.697)	Shoulder	7	Injection therapies	Pain, function	Cochrane	None	Corticosteroid effective in short but not long-term, PRP and prolotherapy superior in the long-term	Yes
**Lin et al. (2018)[45]**	Clin Rehabil (2.738)	Lateral Elbow	6	Botulinum toxin injection (BOTOX)	Pain, function	Cochrane	None	BOTOX injections superior to placebo and as effective as corticosteroid injections (though less effective for short-term pain)	Yes
**Littlewood et**	Physiothera	Shoulder	4	Exercise	Pain,	Cochrane	Cochrane	Evidence	No

**al. (2012)[46]**	py (2.534)				disability	Back Review Group	Back Review Group	supports use of exercise but study largely inconclusive	
**Louwerens et al. (2014)[47]***	J Should Elb Surg (2.865)	Shoulder calcific	20	Minimally invasive therapies	Pain, function, resorption rate	Cochrane	GRADE	High-energy ESWT safe and effective in short- and mid-term	Yes
**Maffulli et al. (2015)[48]**	Br Med Bull (2.804)	Achilles	13	Pharmacological interventions	"Clinical or functional outcomes"	Modified Coleman	None	Inconclusive due to low quality of evidence	No
**Magnussen et al. (2009)[49]**	Clin J Sport Med (2.702)	Achilles	16	Non-operative treatment	Pain, tenderness, tendon thickness, function	None	None	Eccentric exercise has the most evidence for effectiveness. ESWT, injections of corticosteroid, sclerosing agents or deproteinised haemodialysate and topical GTN need more research	No
**Martimbianco et al. (2020)[50]**	Clin Rehabil (2.738)	Achilles	4	Laser therapy	Function, pain, QoL,	Cochrane	GRADE	Inconclusive due to low	Yes

					adverse events			quality of evidence	
**Mendonca et al. (2020)[51]**	Br J Sports Med (11.645)	Patellar	9	Conservative treatment	Pain, function	PEDro	GRADE	Inconclusive due to low quality of evidence	Yes
**Miller et al. (2017)[52]**	BMJ Open Sport Exerc Med (1.510)	All	16	PRP	Pain (not pre-defined)	Cochrane	None	PRP more efficacious than control	Yes
**Mohamadi et al. (2017)[53]**	Clin Orthop Relat Res (4.154)	Shoulder	14	Corticosteroid injections	Pain	Cochrane	None	Corticosteroid injections provide minimal transient pain relief in a small number of patients	Yes
**Murphy et al. (2019)[54]**	Br J Sports Med (11.645)	Achilles	7	Heavy eccentric calf training (HECT)	Pain, function	Cochrane RoB 2	GRADE	HECT may be superior to no treatment and traditional physiotherapy but inferior to other exercise interventions	Yes
**Nogueira & Moura (2015)[55]**	Acta Ortop Bras (0.570)	All	3	Laser therapy	Pain (not pre-defined)	None	None	Laser therapy improves pain in Achilles tendinopathy	No
**Ortega-Castillo**	J Sci Med	Shoulder &	12	Eccentric	Pain,	PEDro	Cochrane	Eccentric	No

**& Medina-Porqueres (2016)[56]**	Sport (3.623)	Lateral elbow		exercise	strength		Back Review Group	exercise effective for pain and strength but its effectiveness compared to other treatments remains questionable	
**Sussmilch-Leitch et al. (2012)[57]**	J Foot Ankle Res (1.604)	Achilles	19	Physical therapies	Pain, function	Modified PEDro, Cochrane	None	Eccentric exercise recommended as first line with or without laser therapy. ESWT may be equally effective	Yes
**Tsikopoulos et al. (2016)[58]**	Phys Ther Sport (2.000)	All	5	PRP	Pain, functional disability	Cochrane	None	PRP provided no more clinical benefit than placebo or dry needling	Yes
**Toliopoulos et al. (2014)[59]**	Clin Rheumatol (2.293)	Shoulder	15	Surgery	Pain, function, ROM (not pre-defined)	Cochrane	None	Surgery no more effective than exercises. Arthroscopic surgery may be superior to open for some	No

								outcome measures	
**Vander Doelen & Jelley (2020)[60]**	J Sci Med Sport (3.623)	Patellar	9	Non-surgical treatments	Pain, function	None	None	Isometric exercise, patellar strapping, sports taping, eccentric exercise, injections with PRP, ABI, and saline and DN demonstrated a short-term pain relieving and functional improvement	No
**Verstraelen et al. (2014)[61]**	Clin Orthop Relat Res (4.154)	Shoulder calcific	5	ESWT	Function, radiological outcomes	Furlan et al. (2009)	None	High-energy more effective than low-energy ESWT for function and deposit resorption	Yes
**Wasielewski & Kotsko (2007)[62]**	J Athl Train (2.253)	Lower Limb tendinopathies	11	Eccentric exercise	Pain, strength	PEDro	None	Eccentric exercise may improve pain and strength	No
**Woodley et al. (2007)[63]**	Br J Sports Med	All	11	Eccentric exercise	Pain, function,	PEDro, Cochrane	Cochrane Back Review	Inconclusive due to low	No

	(11.645)				satisfaction, return to work	Back Review Group	Group	quality of evidence	
**Wu et al. (2017)**[64]	Arch Phys Med Rehabil (2.697)	Shoulder calcific	14	Non-operative treatments	Pain, function, calcification clearance	Cochrane, PEDro	None	US-guided needling and ESWT (radial and high-energy focused) alleviate pain and achieve complete resolution of calcium deposits	Yes
**Xiong et al. (2019)**[65]	Phys Sportsmed (1.874)	Lateral Elbow	4	ESWT vs Corticosteroid	Pain, function	Jadad, Cochrane	None	ESWT may be superior to corticosteroids	Yes
**Yan et al. (2019)**[66]	J Orthop Surg Res (1.907)	Lateral Elbow	5	US therapy and ESWT	Pain, function, pain-free grip strength	Modified Jadad, Cochrane	None	ESWT superior to US therapy up to 6 months for pain and pain-free grip strength	Yes
**Zhang et al. (2019)**[67]	Medicine (Baltimore) (1.870)	Shoulder calcific	8	US-guided lavage	Pain, function, size of calcium deposition, calcification clearance	Cochrane	None	US-guided lavage may be superior to ESWT in pain relief and calcification clearance	Yes

Suppl. Table 1: Characteristics of included systematic reviews and their key findings. ESWT, extracorporeal shock-wave therapy; GTN, glyceryl trinitrate; PRP, platelet-rich plasma; ROM, range of movement; TENS, transcutaneous electrical nerve stimulation; QoL, quality of life; US, ultrasound
*one non-randomised trial included
