# Supplementary material for: Do we need to improve the reporting of evidence in tendinopathy management? A critical appraisal of systematic reviews with recommendations on strength of evidence assessment
Source: BMJ Open Sport Exerc Med. 2021 Feb 23;7(1):e000920. doi: 10.1136/bmjsem-2020-000920 (PMC7907875; doi:10.1136/bmjsem-2020-000920)
Supplement: Supplementary data [file bmjsem-2020-000920supp003.pdf]

Supplementary Figure 2: Useful statistical formulas

***Difference in means (SD) =** $Mean_2 - Mean_1$ $\left(\frac{SD_1 + SD_2}{2}\right)$

$Mean_1$ (SD_1) = Mean (SD) at baseline

$Mean_2$ (SD_2) = Mean (SD) at follow up

* Not the same as mean difference (MD), which is the mean value of the combined differences in means

$$SD_{pooled} = \sqrt{\frac{[SD_1^2(n_1-1)] + [SD_2^2(n_2-1)] + \ldots + [SD_k^2(n_k-1)]}{(n_1 + n_2 + \ldots + n_k - k)}}$$

SD_1 = SD from study 1, SD_2 = SD from study 2 etc

n_1 = sample size from study 1, n_2 = sample size from study 2 etc

k=number of samples/studies

Optimal Information Size (OIS)

$$N = \frac{2(a+b)^2 SD^2}{(x_1 - x_2)}$$

N=the sample size required in each of the groups

$x_1 - x_2$ = minimal clinically relevant difference (defined by authors)

SD^2 = population variance (SD_{pooled} can be used)

a = 1.96 (for 5% type I error)

b = 0.842 (for 80% power)

Required statistic	Available statistic	Conversion
SD	SEM	SD = SEM x $\sqrt{n}$
	IQR	SD = IQR / 1.35
	Range	SD = length of range / 4
	CI	(length of CI / 3.92) x $\sqrt{n}$
95% CI	$Mean_1$ (SD_1) and $mean_2$ (SD_2)	CI = $mean_2$ – $mean_1$ $\pm$ 1.96(SD_{pooled} / n)
Mean	Median	Mean = Median

Useful statistical conversions. CI, confidence interval; IQR, interquartile range; n, sample size; SD, standard deviation; SEM, standard error of mean.
